# Increased spine PIP3 is sequestered from dendritic shafts

**DOI:** 10.1186/s13041-022-00944-5

**Published:** 2022-07-04

**Authors:** Yoshibumi Ueda, Naotoshi Sugimoto, Takeaki Ozawa

**Affiliations:** 1 Department of Chemistry, School of Science, The University of Tokyo, Tokyo, Japan; 2 Department of Physiology, Graduate School of Medical Science, Kanazawa University, Ishikawa, Japan

**Keywords:** Hippocampus, Two-photon microscopy, Phosphatidylinositol 3,4,5-trisphosphate, Fluorescence lifetime, Fluorescence resonance energy transfer, Dendritic spine

## Abstract

Phosphatidylinositol 3,4,5-trisphosphate (PIP3) is a lipid second messenger that is crucial for the synaptic plasticity underlying learning and memory in pyramidal neurons in the brain. Our previous study uncovered PIP3 enrichment in the dendritic spines of hippocampal pyramidal neurons in the static state using a fluorescence lifetime-based PIP3 probe. However, the extent to which PIP3 enrichment is preserved in different states has not been fully investigated. Here, we revealed that PIP3 accumulation in dendritic spines is strictly controlled even in an active state in which PIP3 is increased by glutamate stimulation and high potassium-induced membrane depolarization. Time-course PIP3 analysis clarified the gradual PIP3 accumulation in dendritic spines over days during neuronal development. Collectively, these results deepen our understanding of PIP3 dynamics in dendritic spines, and the dysregulation of the PIP3 gradient between dendritic spines and shafts could cause neuronal diseases and mental disorders, such as autism spectrum disorder.

## Introduction

Phosphatidylinositol 3,4,5-trisphosphate (PIP3) regulates a broad spectrum of cellular functions as a lipid second messenger and is generated by phosphatidylinositol 3-kinase (PI3K) in response to hormones and neurotransmitters. For example, in neuronal cells, PI3K/PIP3 regulates neurite formation [1], the polarity of pyramidal neurons [2, 3], dendritic arborization [4] and axon growth [5, 6]. Additionally, spine PIP3 regulates synaptic function by maintaining α-amino-3-hydroxy-5-methyl-4-isoxazolepropionic acid (AMPA) receptor clustering [7]. To exert these effects, the subcellular distribution of PIP3 is important because PIP3-binding proteins such as Akt [8], WASP family verprolin homologous protein [9] and guanine nucleotide exchange factor small G proteins [10–12] induce signaling by associating with PIP3.

Dendritic spines are thought to be the primary sites of learning and memory in the brain [13]. We previously developed a fluorescence lifetime-based PIP3 probe termed FLIMPA3 [14] and demonstrated PIP3 enrichment within the dendritic spines of CA1 neurons in hippocampal organotypic slices. However, the extent to which PIP3 compartmentalizes into dendritic spines in conditions in which PIP3 is increased remains unknown. Here, we investigated PIP3 dynamics in dendritic spines and shafts in response to glutamate stimulation and membrane depolarization and during neuronal development in hippocampal pyramidal neuronal cells.

## Materials and methods

### Constructs

FLIMPA3 is based on the CFP-YFP ratio metric PIP3 FRET probe Fillip [15]. Monomeric EGFP (mGFP), the FRET donor, was prepared by introducing a single point mutation (A206K) in EGFP. sREACh, the FRET acceptor, was prepared as previously described [16].

### Organotypic hippocampal slice culture

Organotypic slice cultures of the hippocampus were prepared from postnatal Day 6–8 rats in accordance with the animal care and use guidelines. The slices were ballistically transfected after 8–10 days in vitro (DIV) with FLIMPA3. Imaging was performed one day after transfection in the distal part of the main apical dendritic shafts of CA1 pyramidal neurons. Regarding Fig. 4, this experiment was not also conducted with the same neurons during neuronal development because long-term expression of FLIMPA3 could affect the function and structure of dendritic spines.

### Fluorescence lifetime imaging under two-photon microscopy imaging

Slices were maintained in a continuous perfusion of modified artificial CSF (ACSF) containing the following (in mM): 119 NaCl, 2.5 KCl, 3 CaCl_2_, 26.2 NaHCO_3_, 1 NaH_2_PO_4_, and 11 glucose, bubbled and equilibrated with 5% CO_2_/95% O_2_. Regarding Fig. 2, the ACSF solution was replaced to ACSF solution including 50 mM KCl, and then this solution was replaced to original ACSF solution again upon washout process. For Fig. 3, the ACSF solution was replaced to ACSF solution including 20 µM glutamate (0 min) and 1 mM glutamate (16 min). Time-lapse imaging was carried out using a two-photon microscope (Fluoview 1000, Olympus) equipped with a Tsunami laser (Sprectra-Physics) at 910 nm. Epi-fluorescence was detected with a PMT (HPM-100–40; Hamamatsu) that was placed after the wavelength filters (Chroma, HQ510/70-2p for GFP and Brightline multiphoton filter 680SP). Fluorescence lifetime images were produced on a PCI board (SPC-150 N; Becker-Hickl). SPC imaging (Becker-Hickl) was used to generate fluorescence lifetime images. To analyze the fluorescence lifetime value of dendritic spines, a whole region including a neck and body of a dendritic spine was averaged. The fluorescence lifetime value of dendritic shafts was averaged from the base regions (white squares in Figs. 2, 3, 4) of dendritic spines regardless of the edges and centerline of dendrites.

### Statistical analysis

All values are expressed as the mean ± standard error of the mean (S.E.M.). Statistical analyses were performed using Student's t test. Asterisks (*) were used to indicate P values < 0.05, and N.S. indicates no significant difference.

## Results

**Fig. 1:**
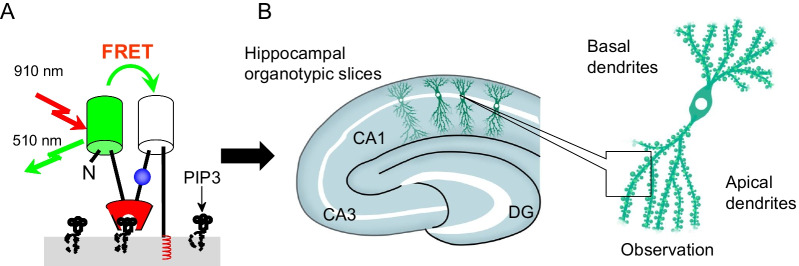
Experimental procedure to observe PIP3 dynamics with FLIMPA3 in hippocampal organotypic slices. **A** Principle of the use of FLIMPA3 to observe PIP3 dynamics. PIP3 production induces a conformational change in FLIMPA3 through the binding of the PH domain to PIP3, leading to an increase in FRET between monomeric GFP (mGFP) and sREARCh. **B** FLIMPA3 was expressed in CA1 pyramidal neuronal cells in hippocampal organotypic slices of rat brains. Then, fluorescence lifetime imaging was performed on dendritic spines and primary and secondary dendritic shafts by two-photon microscopy. DG indicates dentate gyrus

**Fig. 2:**
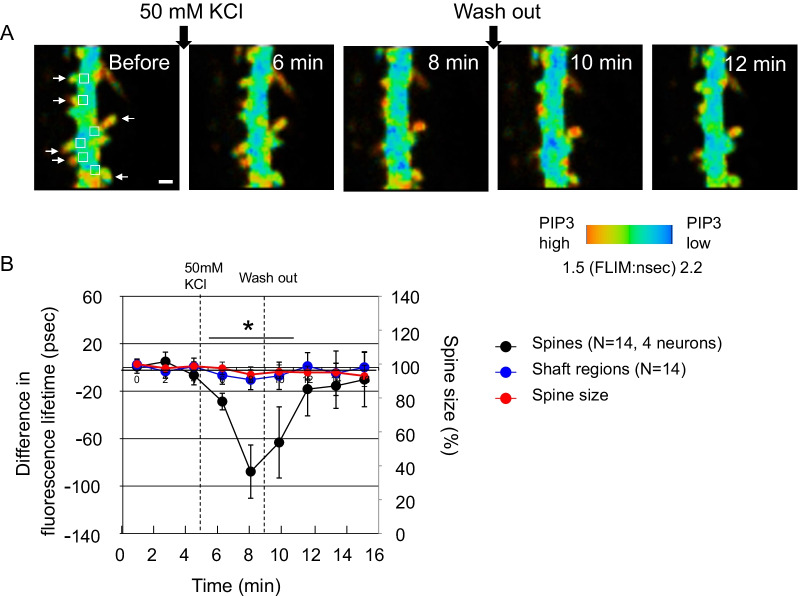
PIP3 is increased in dendritic spines but not dendritic shafts in response to high potassium-induced depolarization. **A** Fluorescence lifetime imaging of FLIMPA3. A color gradient was used to represent PIP3 levels, with a reddish color indicating a shorter fluorescence lifetime and higher PIP3 levels. White arrows indicate dendritic spines. The fluorescence lifetime values of dendritic shafts corresponding to dendritic spines were analyzed from the regions indicated by white rectangles in the image before KCl stimulation. White bar = 1 µm. **B** Time-course analysis of the fluorescence lifetime of dendritic spines and shafts. Asterisks denote a statistically significant difference in the fluorescence lifetime difference between dendritic spines and shafts (p < 0.05)

To visualize spine PIP3, FLIMPA3 [14] (Fig. 1A) was employed. FLIMPA3 was expressed in CA1 pyramidal neurons in hippocampal organotypic slices (Figs. 1B, 2A). The FLIM image showed that the color in dendritic spines was redder than that observed in the dendritic shaft (Fig. 2A), as shown in a previous report [14], indicating that PIP3 was accumulated in dendritic spines but not dendritic shafts.

Potassium chloride (KCl)-induced depolarization through potassium channels induces PI3K signaling activation [17]. Therefore, we investigated the effect of KCl-mediated depolarization on PIP3 dynamics in spines and dendritic shafts. In response to 50 mM KCl, spine PIP3 was quickly increased and then reversibly decreased after KCl was washed out (Fig. 2A and B). The spine size was not changed during this observation (Fig. 2B). Interestingly, during this temporal PIP3 increase, PIP3 at dendritic shafts did not changed (Fig. 2A and B). These data indicate that increased PIP3 was compartmentalized in dendritic spines when PIP3 was increased in dendritic spines.

**Fig. 3:**
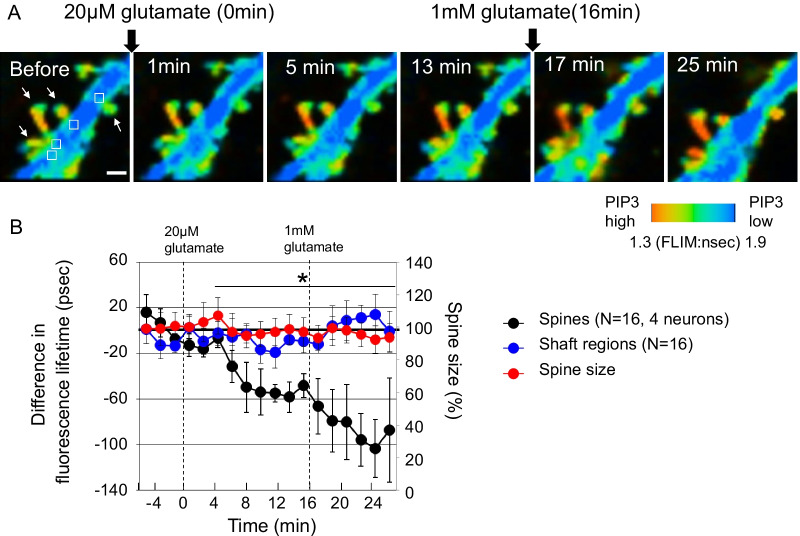
Spatiotemporal PIP3 dynamics at dendritic spines and shafts in response to glutamate administration. **A** Fluorescence lifetime imaging of FLIMPA3 during glutamate administration. White arrows indicate dendritic spines. The fluorescence lifetime values of dendritic shafts corresponding to dendritic spines were analyzed from the regions indicated by white rectangles in the image before glutamate stimulation. White bar = 1 µm. **B** Time course of fluorescence lifetime changes in FLIMPA3 in dendritic spines and dendritic shafts. Asterisks denote a statistically significant difference in the fluorescence lifetime difference between dendritic spines and shafts (p < 0.05)

Next, we examined the effect of glutamate on PIP3 dynamics, since glutamate activates PI3K through metabotropic glutamate receptors [18]. In response to 20 µM glutamate, spine PIP3 gradually increased, and 1 mM glutamate administration induced a further increase in PIP3 (Fig. 3A and B), whereas the spine size was not changed (Fig. 3B). During glutamate addition, PIP3 was not increased in dendritic shafts (Fig. 3A and B), which is consistent with high KCl-induced membrane depolarization and suggests increased PIP3 sequestration from dendritic shafts.

**Fig. 4:**
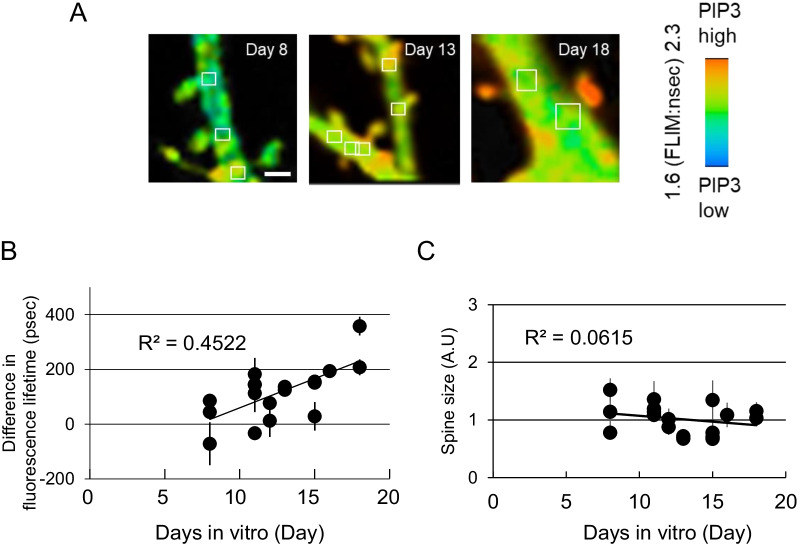
The change in PIP3 levels in dendritic spines and shafts during neurodevelopment. **A** Representative images for each time point during neuronal development. The fluorescence lifetime values of dendritic shafts corresponding to dendritic spines were analyzed from the regions indicated by white rectangles in the image for 8 days. FLIMPA3 was transfected one day before observation in every experiment. White bar = 1 µm. **B** The change in the difference of fluorescence lifetime between dendritic spines and shafts during neuronal development. A total of 110 dendritic spines from 17 neurons were examined. The fluorescence lifetime of the dendritic spines from one neuron was averaged. The values were obtained by subtracting the fluorescence lifetime values of dendritic spines from the fluorescence lifetime values of the corresponding dendritic shafts. A correlation coefficient was calculated by least squares method. **C** The correlation between days in vitro and spine size. The number of spines same with **B** was analyzed

Finally, to investigate when PIP3 accumulation occurs during the process of CA1 neuronal development, we observed the time-dependent effects on PIP3 accumulation in dendritic spines and shafts (Fig. 4). We observed that spine PIP3 is gradually increased through the neuronal development (Fig. 4A). Analysis of the difference in PIP3 between dendritic spines and shafts increased daily (R2 = 0.4522) (Fig. 4B). In contrast, there was no correlation between spine size and neuronal development (R2 = 0.0615)(Fig. 4C), the result of which is consistent with a previous study [19]. Taken together, these data suggest that PIP3 is accumulated during neuronal development.

## Discussion

In the present study, we demonstrated that increased spine PIP3 in excitatory neurons was sequestered from dendritic shafts in the presence of glutamate and KCl stimulation. Additionally, a PIP3 gradient gradually formed during neuronal development. These data suggest that the accumulation of spine PIP3 is strictly controlled in neurons.

We previously demonstrated PIP3 enrichment within the dendritic spines of CA1 neurons using FLIMPA3 [14]. In the present study as well, FLIMPA3 was employed to detect PIP3 at dendritic spines and shafts. As one of advantages using FLIMPA3, because this probe is a membrane-tethered probe, we do not need to consider the background signals from a cytosolic fraction, which becomes a problem for a translocation type of PIP3 fluorescent probes (PH-FP probe), in which a PIP3 binding motif tagged with GFP moves to the plasma membrane upon PIP3 production [19]. Therefore, FLIMPA3 is higher sensitivity than previous PIP3 probes including PH-FP probes. Another advantage using FLIMPA3 in neurons is to allow for easily identifying PIP3 dynamics at dendritic spines and shafts whereas PH-FP probes often lead to misunderstanding of PIP3 signals because spine shape changes affect the fluorescence intensity change in a PIP3-independent manner and cause artifacts. The limitation of FLIMPA3 could induce a toxicity by long-term expression in neurons because the concentration of PIP3 binding motifs is higher at the plasma membrane than that of PH-FP probes. To avoid this issue, FLIMPA3 is transfected one day before observation. The subcellular distribution of signal transduction in neuronal cells plays important roles in a variety of cellular functions, such as long-term potentiation. For example, calmodulin-dependent protein kinase II (CaMkII) [20], Ras [21], cdc42 [22], RhoA [22], and Rac [23] are activated in dendritic spines in response to glutamate stimulation. While CaMkII activation is restricted to dendritic spines [20], Ras signaling spreads to dendritic shafts and invades neighboring dendritic spines, which affects the functional and structural plasticity of neighboring dendritic spines[21]. In the present study, we demonstrated that increased PIP3 was sequestered from dendritic shafts in conditions in which PIP3 was increased (Figs. 2 and 3). There are mainly two possibilities for increased PIP3 accumulation at the dendritic spines. The first possibility is due to the difference in the subcellular distribution of PI3K and PTEN. PTEN is localized in dendritic shafts [24], whereas active PI3K accumulates in dendritic spines [25]. Considering this evidence, the PIP3 that moved to dendritic shafts could be quickly erased by PTEN at dendritic shafts. The other possibility is that barrier structures may exist to hinder the lateral movement of PIP3 at the spine necks. In Fig. 2, after KCl was washed out, PIP3 level returned to the baseline. This phenomenon could be explained by the former possibility. However, we cannot exclude another possibility that PIP3 movement to dendritic shafts is stopped by barrier structures at the spine necks, and the PIP3 was degraded by a few amounts of PTEN localized at dendritic spines.

PTEN deficiency and mutations that cause persistent activation of PI3K signaling induce autism spectrum disorder-like behaviors [26]. Considering our results, PTEN deficiency could cause PIP3 efflux to dendritic shafts and affect a synaptic plasticity of neighbor dendritic spines. Further investigation is needed to determine whether downstream components of PI3K signaling, including Akt, mTOR, and eIF4E, are also activated at only dendritic spines.

Further investigation will be needed to reveal the mechanism underlying spine PIP3 accumulation through neuronal development in Fig. 4. However, spine activations induced by stimulations with KCl (Fig. 2) and glutamate (Fig. 3) increased spine PIP3. These repeated spine activations could contribute to gradual PIP3 accumulation in dendritic spines through neuronal development.

Subspine PIP3 distribution could play an important role in spine morphology. Previously, we discovered that spine PIP3 regulates spinule formation on dendritic spines [14] in response to long-term potentiation using glutamate uncaging methods [27]. In the present study, we often observed uneven subspine PIP3 localization, in which PIP3 was concentrated at spine necks more than in spine tips in several dendritic spines (Fig. 3A). Active PI3K was localized in spine tips with a postsynaptic density [25]. Therefore, PIP3 could move to spine necks after being produced by PI3K at spine tips.

In conclusion, the present study showed increased PIP3 sequestration from dendritic shafts in any condition in which PIP3 was increased, which contributes to a deeper understanding of spine PIP3 dynamics.

## Data Availability

Data sharing not applicable to this article as no datasets were generated or analysed during the current study.
